# Awareness, Attitudes, and Concerns Regarding Heated Tobacco Products Among Physicians in Japan

**DOI:** 10.2188/jea.JE20210470

**Published:** 2023-09-05

**Authors:** Yuichiro Otsuka, Yoshitaka Kaneita, Osamu Itani, Yuuki Matsumoto, Yutaka Hatori, Satoshi Imamura

**Affiliations:** 1 Division of Public Health, Department of Social Medicine, Nihon University School of Medicine, Tokyo, Japan; 2 Japan Medical Association, Tokyo, Japan

**Keywords:** heated tobacco product (HTP), Japan, physician, smoking

## Abstract

**Background:**

New tobacco products, particularly heated tobacco products (HTPs), have been introduced across several international markets as alternatives to combustible products, such as cigarettes. However, there are limited studies on physicians’ perceptions of HTPs. This study analyzes the awareness of HTPs among physicians and assesses their concerns and attitudes toward patients using HTPs.

**Methods:**

A self-administered questionnaire was sent to a sample of 7,500 member physicians (6,000 male and 1,500 female) from the Japan Medical Association (JMA). The smoking status of physicians was categorized as never smokers, ever HTP smokers, current HTP smokers, and ever and current smokers of other products. Physicians’ awareness and attitudes toward patients using HTPs were analyzed using descriptive statistics. The correlation between the outcomes was examined using logistic regression models, whereas physicians’ concerns about HTPs were analyzed using descriptive statistics.

**Results:**

Data were obtained from 5,492 physicians (21.9% women; aged 60.4; standard deviation, 12.1 years) with a response rate of 74.6%. Overall, 76.7% of the physicians had awareness of HTP, and about half of whom asked patients about using HTPs. Physicians who took longer to discuss cessation were more likely to discourage patient use. Smoking status was associated with discouraging HTP use in patients. Physicians who had experience with HTP use were primarily concerned about the products’ long-term safety and less about product regulation.

**Conclusion:**

Japanese physicians do not have sufficient information and knowledge about HTPs. Therefore, evidence-based guidelines are required to support physicians in advising patients against HTP use.

## INTRODUCTION

Heated tobacco products (HTPs), also known as “heat-not-burn” products, are electronic devices that heat tobacco to produce an aerosol (as opposed to cigarette smoke).^1^ In 2014, HTPs were introduced across Japan; thereafter, they were introduced across Italy and Switzerland in 2015. Since then, HTPs have gained widespread prevalence in more than 60 markets across Europe, North and South America, Eurasia, and East Asia.^2^ Japan was the first country globally to sell IQOS, a major HTP brand. In 2016, Japan accounted for 96% of the global share of IQOS sales.^3^ Subsequently, the Pharmaceutical Affairs Act prohibited the sale of nicotine e-cigarettes. This may have influenced the public into thinking that HTPs are not a type of electronic cigarette or smoking product. An Internet-based survey indicated that HTP users in Japan increased from 0.2% in 2015 to 11.3% in 2019 among participants aged 15–69 years.^4^ A Japanese population-based survey reported that the age-adjusted prevalence of lifetime HTP smokers was 14.1% in men and 3.7% in women.^5^ The age-adjusted prevalence for current HTP smokers was 8.3% in men and 1.9% in women.^5^ Users rated HTPs as less satisfying than conventional cigarettes^1^^,^^6^ and puffed more frequently than the specified doses, even though their nicotine levels are comparable to conventional cigarettes.^7^^,^^8^ However, a systematic review reported that HTPs have an increased prevalence among young adults, including nonsmokers in many countries.^9^

HTPs have been considered less harmful alternatives to conventional cigarettes.^1^^,^^8^^,^^10^ However, HTPs have similar nicotine concentrations to conventional cigarettes, which triggers nicotine addiction,^11^ making cessation difficult.^12^ HTP use can encourage conventional cigarette use.^13^^,^^14^ Public health experts found that HTPs continue to pose a health risk for smokers and nonsmokers because they contain nicotine, carcinogens, and heavy metals that are released with the exhaled steam.^6^^,^^15^ Several case reports have revealed an association between HTPs and the onset of acute eosinophilic pneumonia.^16^^,^^17^ Thus, even though the health effects of short- and long-term HTP use remain unclear, the dual use of conventional cigarettes and HTPs does not reduce the harmful effects of substance exposure.

Physicians have often been deemed role models regarding health behavior and play a principal role in smoking-cessation practices.^18^^,^^19^ Due to the exponential growth of HTP awareness and use, coupled with aggressive marketing by tobacco companies, physicians have increased their engagement with patients who smoke cigarettes smoking about HTP use. However, many physicians seem unprepared to provide informed and consistent advice to patients and families. The confusion was escalated by the United States Food and Drug Administration’s decision about IQOS’s status under its “modified risk tobacco product” criteria.^20^ The tobacco industry has associated HTPs with “harm reduction,” branding them as a more socially acceptable cigarette. Therefore, physicians need to have the necessary HTP knowledge and provide health-related guidance.

Despite the growing number of HTP markets, little is known about physicians’ awareness, attitudes, and concerns toward HTP use in patients. In a 2019 survey of 322 Turkish family physicians, only 9.0% knew of IQOS and 83.9% had no opinion.^21^ This study was limited by a relatively small sample size and a skewed representation of the study population. Previous studies suggest that physician smoking status was related to smoking cessation counseling.^22^^,^^23^ Thus, it was hypothesized that physicians who smoke have unfavorable beliefs and attitudes and more perceived barriers to discussing the use of HTPs than those who do not. Concurrently, smoking physicians would be more accepting of people’s use of HTPs. This study aimed to explore the general awareness, attitudes, and concerns of physicians by smoking status history using a large nationwide sample. Tobacco companies have been aggressively marketing HTPs in Japan, and many smokers now tend to use HTPs alone or in combination with conventional cigarettes.^5^^,^^24^ Meanwhile, Japan continues to receive poor ratings for its tobacco-control measures.^25^ The current study is, therefore, expected to provide important evidence for public health-related campaigns and regulations of HTPs in other countries that allow their sale.

## METHODS

### Participants and procedures

Since 2000, the Japan Medical Association (JMA) and our study group jointly began conducting epidemiological surveys of the smoking behavior of JMA members. This survey employed data collected in 2020. Participants comprised physicians (6,000 men and 1,500 women) randomly selected from among 172,763 JMA members on December 1, 2019. A systematic sampling approach for random selection was adopted. First, a roster listing the medical registration number and gender was created. Second, the first survey target was randomly selected, and the second and subsequent survey targets were extracted at regular intervals. This method did not show age or regional bias.^22^ Approximately 60% of all Japanese physicians are members of the JMA.

A package containing four items was posted to the target population by mail. Each package contained a self-administered questionnaire, a letter requesting cooperation to survey participants’ smoking behavior, a medium-sized return envelope, and a small envelope for enclosing the questionnaire. Participants were asked to enclose the filled-in questionnaire in a small envelope and seal it, then put it in a medium-sized envelope to return it to the JMA. To identify those who did not return questionnaires, the medium-sized return envelope had a label bearing the participant’s name and address. The questionnaire and small envelope were not labeled. A second package was sent to the target population who had not returned the questionnaire, again asking for their cooperation. This follow-up procedure was repeated up to three times. The survey period was January to August 2020.

The ethics committee of JMA and Nihon University School of Medicine approved this study, which was conducted in accordance with the Declaration of Helsinki and ensured participants’ confidentiality (No. R1-7).

### Sample size

The sample size was determined using the statistic corresponding to the level of confidence, expected prevalence, and precision (corresponding to effect size).^26^ Thus, we assumed 70% for the prevalence of HTP awareness based on a previous Japanese survey and set the precision of 1.0%. Consequently, the sample size was estimated to be at least 1,896 participants.

### Measures

#### Definition of tobacco products

We defined tobacco products as conventional cigarettes, cigars, chewing tobacco, snuff, HTPs, and e-cigarettes. To distinguish between HTPs and other products, we used the most popular brands of HTPs in the questionnaire, including IQOS, glo, and Plume Tech.

#### Smoking status

Physicians’ smoking status was classified using the following categories based on previous surveys: *never smokers* have never smoked any tobacco products; *ever non-HTP smokers* had smoked tobacco products except HTPs, but had not smoked in the past 30 days; *current non-HTP smokers* had smoked tobacco products except HTPs in the past 30 days; *ever HTP smokers* had smoked HTPs, but had not smoked HTPs in the past 30 days; and *current HTP smokers* had smoked HTPs in the past 30 days.^14^^,^^27^ These categories prioritized the smoking status of HTP as compared to the other tobacco products. For example, even if physicians smoked tobacco products other than HTP in the past 30 days, and had used HTP prior to these 30 days, they were classified as ever HTP smokers. If physicians smoked HTP in the past 30 days regardless of using other tobacco products, those were classified as current HTP.

#### HTP awareness

To measure HTP awareness, participants were asked, “Are you aware of HTPs and e-cigarettes?” to which they could respond with (1) I am not aware of either HTPs or e-cigarettes; (2) I am aware of e-cigarettes, but not HTPs; (3) I am aware of HTPs, but not e-cigarettes; or (4) I am aware of HTPs and e-cigarettes. Participants who responded using options (3) or (4) were deemed to have HTP awareness.

#### Physicians’ inquiry regarding and advice against HTPs

Responses about HTP use were dichotomized into “yes” or “no.” Physicians’ advice concerning HTP use to patients was assessed from patients asking physicians’ advice to using HTP. Respondents choose one of three options: “No, you should not use them,” “Yes, you can use them as they are useful as a substitute for cigarettes,” and “I do not know if you can use it or not.”

#### Physicians’ HTP concerns

We asked physicians with awareness of HTP about 11 items regarding individual HTP concerns that were divided into three categories: (1) health risks, (2) addictive potential, and (3) regulation.

### Covariates

We determined physicians’ attitude toward smoking and the cessation discussion time they spent with their patients (>5 minutes or more, 3–5 minutes, <3 minutes, and none). The survey collected personal and professional demographics (eg, sex, age, specialty, employment type, and work facility).

### Statistical analyses

Calculations were standardized according to the age group and sex composition of physicians provided by Japan’s Ministry of Health, Labour and Welfare,^28^ and all the calculations were weighted. The main outcome was the proportion of HTPs awareness. Missing data were imputed using multiple imputation by chained equations.^29^ It is usually performed under the missing at random assumption; however, it may also be performed under specific missing not-at-random assumptions.^29^ Complete data (sex, age group, and smoking status) were used to predict the missing values (cessation discussion time, medical department, employment type, and facility) using ordinal and multinomial logistic models. A total of 20 datasets were produced for each of the multiple imputation models. Analyses of the imputed data provided essentially the same results as those from complete case analyses. A one-way analysis of variance (ANOVA) was performed to compare awareness of HTPs by demographics and other basic information. We then conducted multivariate logistic regression analyses to explore HTP awareness, with an odds ratio calculation. In multivariate logistic regression models, we adjusted for sex, age group, smoking status excluding experienced HTP smokers, cessation discussion time, medical department, employment type, and facility. To assess the robustness of our results, we reanalyzed “HTP awareness” among physicians who were aware of HTPs and e-cigarettes as sensitivity analysis. Third, for physicians who were aware of HTP, we examined differences in physicians’ attitudes toward HTPs by smoking status using ANOVA. Fourth, among physicians who were aware of HTPs, unadjusted and multivariate logistic regression analyses were conducted for whether physicians believed that patients should not use HTPs. The adjusted variables were sex, age group, smoking status, cessation discussion time, medical department, employment type, facility, and concerns about HTP. To avoid multicollinearity in multivariate logistic regression, this study used the Spearman correlation coefficient to investigate the correlation between all concerning matters regarding HTP. Items with coefficient values over 0.4 were deleted. The differences in HTP concerns among those with HTP awareness were calculated from the smoking status using ANOVA. Statistical analyses were conducted using Stata version 16.0 (StataCorp, College Station, TX, USA). The significance level was set to 0.05.

## RESULTS

### Participants

The study employed a cross-sectional design, using the data from “The 2020 Survey on Smoking and Related Factors.” Questionnaires were sent to 7,500 participants; 137 did not receive the survey and were excluded owing to factors such as unknown address, hospitalization, or death. Of the remaining 7,363, there were 5,591 responses. Of these, 99 were excluded for incomplete data, and 5,492 participants were included (response rate 74.6%). Table 1 shows participants’ characteristics. The average respondent age was 60.4 (standard deviation, 12.1) years (21.9% women); 49.8% majored in internal medicine. Among the physicians, 2.6% (95% confidence interval [CI], 1.9–3.5%) were current HTP smokers, 0.8% (95% CI, 0.5–1.3%) were ever HTP smokers, and 3.5% (95% CI, 2.8–4.2%) were current non-HTP smokers. Cessation discussion time was missing in 1.9% of cases, medical department was missing in 0.3%, work form was missing in 3.2%, and facility was missing in 4.4%.

**Table 1.** The characteristics of the participants in this study (*N* = 5,492)

	Participants			
	
	Unweighted, Number	Weighted %	95% CI	
Sex				
Male	4,344	78.1		
Female	1,148	21.9		
Age group, years				
24–39	244	29.4		
40–49	859	21.4		
50–59	1,486	21.6		
60–69	1,622	17.2		
≥70	1,281	10.5		
Smoking status				
Never-smoker	3,712	72.6	70.9	74.2
Ever non-HTP smoker	1,412	20.6	19.3	22.0
Current non-HTP smoker	228	3.5	2.8	4.2
Ever HTP smoker	39	0.8	0.5	1.3
Current HTP smoker	101	2.6	1.9	3.5
Cessation discussion time				
None	1,379	21.0	19.5	22.6
<3 min	2,342	48.6	46.5	50.8
≥3 min, <5 min	1,096	20.9	19.2	22.8
≤5 min	572	9.4	8.4	10.6
Medical department				
Internal medicine	2,710	49.8	47.6	51.9
Surgery	303	5.4	4.5	6.4
Orthopedics	377	6.4	5.5	7.4
Pediatrics	323	5.6	4.7	6.7
Gynecology	285	5.8	4.8	6.9
Psychiatry	220	4.5	3.6	5.5
Dermatology	210	2.9	2.4	3.4
Urology	114	2.2	1.6	2.9
Ophthalmology	316	6.0	5.1	7.2
Otolaryngology	229	4.2	3.4	5.1
Medical examination	21	0.3	0.2	0.6
Other	368	7.0	6.0	8.1
Work form				
Owner	2,919	44.4	42.6	46.3
Employee	2,400	55.6	53.7	57.4
Facility				
Clinics	3,235	53.3	51.2	55.4
Hospitals	1,738	43.4	41.3	45.5
Other	277	3.4	2.9	3.9

CI, confidence interval; HTP, heated tobacco product.

There is missing data on the cessation discussion time from 103 respondents (1.9%), medical department data from 16 respondents (0.3%), work form data from 173 respondents (3.2%), and facility data from 242 respondents (4.4%).

### HTP awareness

Table 2 shows the percentage and factors of each physician’s awareness of HTP. Overall, 76.7% (95% CI, 75.3–78.1%) of the physicians had HTP awareness. Younger physicians and current non-HTP smokers were more familiar with HTPs. Among the medical departments, physicians specializing in internal medicine were most aware of HTPs (79.2%; 95% CI, 77.2–81.2%), while surgery and orthopedics were least aware (73.0%; 95% CI, 68.8–77.1%). The odds ratios of HTP awareness were significantly lower in women (adjusted odds ratio [aOR] 0.70; 95% CI, 0.52–0.86) than men; in older participants, especially those over 70 years (aOR 0.10; 95% CI, 0.06–0.16); and in surgery and orthopedics specialties (aOR 0.78; 95% CI, 0.59–0.98), as compared to internal medicine. The odds ratios of HTP awareness were significantly higher in current non-HTP smokers (aOR 1.83; 95% CI, 1.31–2.57) than never smokers, as well as physicians providing guidance on smoking-cessation to patients than their counterparts.

**Table 2.** Rate and odds ratio of physicians’ awareness about HTP (*N* = 3,730)

	Unweighted, No	Weighted (%, 95% CI)^*a^		*P*-value^*b^	aOR	95% CI	*P*-value^*c^
Total	3,730	76.7	(75.3–78.1)				
Sex							
Male	2,964	76.8	(75.3–78.3)	0.591	1.00		
Female	766	76.4	(72.9–79.5)		0.66	(0.52–0.86)	0.002
Age group, years							
24–39	221	90.9	(86.6–94.0)	<0.001	1.00		
40–49	681	80.2	(77.4–82.7)		0.39	(0.24–0.64)	<0.001
50–59	1,101	74.5	(72.2–76.7)		0.27	(0.17–0.44)	<0.001
60–69	1,074	66.4	(64.1–68.7)		0.18	(0.11–0.29)	<0.001
≥70	653	51.2	(48.4–53.9)		0.10	(0.06–0.16)	<0.001
Smoking status							
Never smoker	2,484	76.4	(74.6–78.1)	<0.001	1.00		
Ever non-HTP smoker	941	73.3	(70.8–75.8)		1.17	(0.99–1.38)	0.073
Current non-HTP smoker	165	80.4	(75.5–84.5)		1.83	(1.31–2.57)	<0.001
Ever HTP smoker	39				—		
Current HTP smoker	101				—		
Cessation discussion time							
None	800	64.7	(61.2–68.2)	<0.001	1.00		
<3 min	1,609	78.5	(76.5–80.6)		1.50	(1.21–1.86)	<0.001
≥3 min, <5 min	841	83.1	(80.3–85.8)		2.19	(1.66–2.88)	<0.001
≤5 min	432	80.0	(75.6–84.4)		2.04	(1.46–2.87)	<0.001
Medical department							
Internal medicine	1,927	79.2	(77.2–81.2)	<0.001	1.00		
Surgery & orthopedics	436	73.0	(68.8–77.1)		0.78	(0.59–0.98)	0.046
Other	1,357	74.6	(72.1–77.1)		0.88	(0.71–1.08)	0.209
Work form							
Owner	1,969	72.7	(70.6–74.7)	<0.001	1.00		
Employee	1,681	79.9	(78.0–81.9)		1.02	(0.76–1.31)	0.895
Facility							
Clinics	2,196	73.8	(71.9–75.8)	<0.001	1.00		
Hospitals	1,222	81.0	(78.8–83.2)		1.03	(0.77–1.38)	0.841
Other	169	67.5	(61.1–74.0)		1.17	(0.82–1.67)	0.383

aOR, adjusted odds ratio; CI, confidence interval; HTP, heated tobacco product.

^*a^Weighted percentages were standardized according to the age group and sex composition of physicians—as provided by Japan’s Ministry of Health, Labour and Welfare.

^*b^*P*-values were calculated by the analysis of variance.

^*c^*P*-values were calculated by multivariate logistic regression.

^d^Odds ratios were adjusted for participants and statistics: population weight, sex, age group, smoking status, cessation discussion time, medical department, employment type, and facility.

^e^Data was missing, namely for cessation discussion time from 48 respondents (1.29%), medical department from 10 respondents (0.27%), work form data from 80 respondents (2.14%) as well as facility data from 143 respondents (3.83%).

^f^Missing data were imputed using multiple imputation by chained equations of 20 datasets.

The sensitivity analysis showed that the proportion and odds ratios of awareness of HTP and e-cigarettes were the same as the original results (eTable 1). Overall, 67.2% (95% CI, 65.3–68.9%) of the physicians had awareness of HTP and e-cigarettes. Physicians younger than current HTP smokers were more familiar with HTP and e-cigarettes. Among medical departments, physicians specializing in internal medicine were most aware of HTPs (70.3%; 95% CI, 67.8–72.8%), whereas surgery and orthopedics were the least aware (60.9%; 95% CI, 55.4–66.4%). The odds ratios of HTP and e-cigarettes awareness were significantly lower among women (aOR 0.70; 95% CI, 0.54–0.89); in older participants, particularly over 70 years (aOR 0.16; 95% CI, 0.11–0.24); and in surgery and orthopedics specialties (aOR 0.74; 95% CI, 0.55–0.99), in contrast to internal medicine. The odds ratios of HTP and e-cigarettes awareness were significantly higher for physicians who provided smoking-cessation guidance to patients than those who did not, suggesting robust awareness of HTP among physicians. The goodness-of-fit tests were not significant, which indicated good model fit.

### Physicians’ attitudes toward patient HTP use

Table 3 shows physician attitudes toward patient HTP use among those with HTP awareness (*N* = 3,730). About a half of the physicians asked patients about using HTPs, while ever HTP smokers had the lowest rate (36.1%). These differences were not significant based on smoking status. Patients using HTPs, most never smokers (80.4%) and ever non-HTP smokers (78.8%) discouraged the use of HTP, whereas current HTP smokers (52.6%), ever HTP smokers (48.3%), and current non-HTP smokers (43.4%) were less likely to agree with this opinion; thus, the physicians were indecisive. In addition, 2.3% of all physicians agreed on their use as a substitute for conventional cigarettes. Current HTP smokers (9.9%) were more prevalent than other smoking status.

**Table 3.** Physicians’ attitudes toward HTPs by smoking status among those with HTP awareness (*N* = 3,730)

Physicians’ briefs and attitudes toward smoking		Total (*N* = 3,730)		Never smoker (*N* = 2,484)		Ever non-HTP smoker (*N* = 941)		Current non-HTP smoker (*N* = 165)		Ever-use HTP smoker (*N* = 39)		Current HTP smoker (*N* = 101)		F-statistic	*P*-value
Asking patients about their HTP use															
	Yes	49.8	(47.2–52.4)	49.9	(46.7–53.2)	49.1	(44.6–53.7)	50.0	(37.9–62.0)	36.1	(19.5–56.7)	54.8	(39.5–69.3)	0.5	0.721
What was your response when a patient asked you about using HTPs?															
1	No	77.4	(75.2–79.5)	80.4	(77.7–82.8)	78.8	(74.6–82.4)	43.4	(31.7–55.9)	48.3	(27.1–70.1)	52.6	(37.6–67.2)	9.1	<0.001
2	Yes	2.3	(1.7–3.2)	1.7	(1.1–2.6)	2.9	(1.4–5.6)	4.1	(2.0–8.1)	4.5	(1.3–14.0)	9.9	(3.3–25.7)		
3	I do not know	16.0	(14.1–18.0)	14.5	(12.3–17.0)	12.8	(10.2–16.1)	37.8	(26.6–50.6)	44.6	(23.6–67.7)	33.0	(21.5–47.0)		
4	Other	4.3	(3.5–5.3)	3.4	(2.6–4.5)	5.6	(3.6–8.5)	14.7	(7.4–26.9)	2.6	(0.62–10.4)	4.5	(1.8–10.8)		

CI, confidence interval; HTP, heated tobacco product.

^a^Data are presented as percentages.

^b^Percentages were standardized according to the age group and sex composition of physicians provided by Japan’s Ministry of Health, Labour and Welfare.

^c^*P*-values were calculated by analysis of variance.

^d^1,762 respondents were not aware of HTP, as such were excluded from this analysis.

Multivariate analyses indicate that physicians who spent more time on cessation discussions were more likely to discourage their patients from the use of HTP (eTable 2). This was observed, with higher rates among internal medicine physicians than other specialists, except for surgery and orthopedic physicians. Increased odds of discouraging HTP use were associated with physicians who agreed that “misguided assumptions that HTPs are less harmful than cigarettes,” “long-term health effects of nicotine addiction,” and “there is a lack of regulatory controls in the government.” Conversely, current and ever HTP smokers were less likely to discourage HTP use than never smokers. The goodness-of-fit tests were not significant, thus indicating a good model fit.

### Physicians’ concerns about HTPs

Table 4 shows HTP use concerns among physicians with HTP awareness (*N* = 3,730). Regarding health effects, most participants (69.0%) agreed with the statement about a lack of evidence regarding their long-term safety, and almost all experienced HTP smokers (ever and current HTP smokers) agreed. However, experienced HTP smokers were less likely to agree with the statements “misguided assumption that HTPs are less harmful than cigarettes” and “misguided assumption that HTP does not causing passive smoking.”

**Table 4.** HTP concerns among physicians with HTP awareness (*N* = 3,730)

		Total (*N* = 3,730)		Never smoker (*N* = 2,484)		Ever non-HTP (*N* = 941)		Current non-HTP (*N* = 165)		Ever HTP (*N* = 39)		Current HTP (*N* = 101)		F-statistic	*P*-value
					
		%	(95% CI)	%	(95% CI)	%	(95% CI)	%	(95% CI)	%	(95% CI)	%	(95% CI)		
Health effects of HTPs															
	Lack of evidence regarding the long-term safety of HTPs	69.0	(66.6–71.3)	68.1	(65.1–71.0)	68.8	(64.6–72.7)	63.6	(51.0–74.7)	91.8	(78.8–97.2)	87.6	(72.9–94.9)	3.95	0.005
	Misguided assumption that HTPs are less harmful than cigarettes	52.4	(49.8–54.9)	54.2	(51.0–57.4)	54.4	(49.9–58.9)	35.6	(25.3–47.3)	26.6	(13.7–45.3)	26.6	(15.1–42.4)	7.58	<0.001
	Misguided assumption that HTP does not causing passive smoking	42.0	(39.5–44.6)	44.3	(41.1–47.5)	39.4	(35.1–43.8)	28.7	(19.2–40.4)	30.0	(15.8–49.5)	25.9	(13.6–43.8)	3.66	0.009
Addictive potential of HTPs															
	Long-term health effects of nicotine addiction	38.9	(36.4–41.5)	39.9	(36.8–43.1)	42.7	(38.2–47.3)	18.5	(12.5–26.4)	17.2	(8.2–32.9)	25.5	(14.2–41.5)	6.47	<0.001
	HTP use may perpetuate smokers’ addiction	23.5	(21.4–25.7)	24.2	(21.6–27.0)	25.2	(21.5–29.2)	10.0	(6.2–15.9)	13.4	(5.3–30.3)	16.8	(7.1–34.5)	2.54	0.057
	HTP use may result in combined cigarette usage	7.5	(6.4–8.9)	7.8	(6.3–9.6)	8.1	(6.4–10.1)	5.1	(2.5–10.2)	6.5	(1.8–20.5)	2.1	(0.62–7.2)	1.55	0.186
Regulation of HTPs															
	Lack of regulatory controls from the government	29.4	(27.2–31.7)	31.9	(29.1–34.9)	27.5	(23.9–31.5)	13.3	(8.5–20.2)	8.4	(2.8–22.5)	11.0	(4.1–26.5)	7.38	<0.001
	Function as attractive starter products and a gateway to smoking among young nonsmokers	40.2	(37.7–42.8)	42.9	(39.8–46.2)	38.2	(33.9–42.6)	30.5	(19.8–43.8)	14.8	(6.3–31.0)	12.7	(5.2–27.7)	7.47	<0.001
	Marketing and advertising of HTPs, especially to children and youth	24.2	(22.0–26.5)	26.7	(23.9–29.7)	21.2	(17.8–25.1)	15.2	(7.8–27.5)	5.2	(1.2–20.0)	4.3	(1.8–10.1)	7.92	<0.001
	HTP functioning as “bridge product” in places where smoking is prohibited	30.4	(28.1–32.8)	31.9	(29.0–34.9)	31.6	(27.6–35.8)	26.5	(16.2–40.2)	11.2	(4.4–25.5)	3.2	(1.2–8.2)	8.41	<0.001
	Advertisement of HTPs by celebrities who vape will make cigarette smoking glamorous again and “renormalize” smoking	21.2	(19.2–23.5)	23.6	(20.9–26.5)	18.0	(15.0–21.5)	9.3	(5.5–15.2)	3.3	(0.46–20.5)	7.9	(2.1–25.8)	5.32	0.002

CI, confidence interval; HTP, heated tobacco product.

^a^Data are presented as percentages.

^b^Percentages were standardized according to the age group and sex composition of physicians provided by Japan’s Ministry of Health, Labour and Welfare.

^c^*P*-values were calculated by analysis of variance.

^d^1,762 respondents were not aware of HTP as such were excluded from this analysis.

Regarding the addictive potential, ever non-HTP smokers reported the highest agreement with these statements. Nearly one-fourth agreed that “HTP use leads to long-term health effects of nicotine addiction.” Only 2.1% of current HTP smokers agreed with the statement “HTP users would be dual cigarette users.” Only 10.0% of all current non-HTP smokers agreed with the statement “HTP use may perpetuate smokers’ addiction.” However, the latter two did not have a significant association with smoking status.

Regarding regulation, 40.2% agreed that HTPs are attractive starter products and a gateway to smoking for young nonsmokers. Never smokers agreed the most with all statements. However, experienced HTP smokers agreed less with regulation statements. These five statements had a significant association with smoking status.

A relatively strong correlation was observed between “misguided assumptions that HTPs are less harmful than cigarettes” and “misguided assumptions that HTPs do not cause passive smoking.” We obtained the same result between “long-term health effects of nicotine addiction” and “HTP use may perpetuate smokers’ addiction,” and among most of the regulations of HTPs (eTable 3).

## DISCUSSION

To the best of our knowledge, this is the first large-scale study aiming to assess awareness, attitudes, and concerns for HTP use among physicians representing various medical specialties in Japan. The main findings of this study are that: (1) most physicians had HTP awareness and agreed that there is a lack of evidence regarding their long-term safety; (2) only half of the physicians discouraged HTP use in patients; (3) only a few physicians believed that HTPs are useful as a conventional cigarette substitute; and (4) physicians’ smoking status and cessation behaviors largely affected their attitudes toward patients’ HTP use and concerns.

HTP awareness in Japanese physicians (76.7%) was higher than in the Japanese general population aged 15–69 years (48% in 2015). However, considering the rapid spread of HTP use in Japan,^4^ there seemed to be no difference in HTP awareness between physicians and the general population.^21^ Few studies that surveyed HTP awareness have targeted medical professionals. A cross-sectional study targeted 1,344 Polish medical students in 2019 and reported that 76.5% of the respondents had HTP awareness.^30^ Furthermore, compared to the Turkish study with family physicians, where HTP use is limited, Japanese physicians may not have adequate awareness regarding HTPs.

Currents smokers were more aware than never smokers among Japanese physicians, and similar results were observed in Japan,^27^ the United States,^31^ and Europe.^32^ The estimated prevalence of current smoking among Japanese physicians was 6.1% in this study, while in 2018, it was 19.0% in the Japanese general population.^5^ Interest in trying HTPs was associated with conventional cigarette and e-cigarette use.^33^ Since physician never smokers know smoking is harmful, they would be less interested in HTPs.

Young people and men had more awareness of HTP, in line with previous research.^27^^,^^31^^,^^32^ Physicians who majored in internal medicine may acquire HTP knowledge from the “Views and Recommendations on HTPs and E-Cigarettes,” published by the Japanese Respiratory Society.^34^ However, other specialists may be less interested due to its indirect relevance to their clinical practice. Physicians who carefully instruct patients to cease smoking may be highly interested in its effects; thus, they are likely highly motivated to acquire HTP knowledge. The results suggested that there was a large variation in HTP awareness among physicians. In the future, there should be better efforts to educate all physicians on HTPs and their dangers.

Only half of the physicians with HTP awareness asked whether their patients smoke HTPs. Several factors may influence this. First, there was a lack of HTP knowledge, unlike that regarding conventional cigarettes. Second, some physicians may consider smoking cessation treatment as time-consuming and ineffective. Third, physicians may not be satisfied with the financial reward of cessation treatment.

When the tobacco industry markets HTPs as less harmful than conventional cigarettes, it may attract the health-conscious population.^35^ By contrast, the actual health impacts on users and public health are not yet fully understood.^36^ Thus, the Japanese Respiratory Society stated that physicians should not recommend HTP use to patients, and most physicians considered discouraging it. However, 10% of physicians who are current HTP smokers regarded HTPs as a useful tool and substitute for cigarettes. Physicians’ behaviors toward smoking-cessation intervention were associated with their smoking status.^22^ Physicians who regarded HTPs as a harm-reduction or smoking-cessation tool may have based this belief on tobacco company advertisements or their own experience. There is no scientific evidence about whether HTP use can reduce harm or help people quit smoking. Contrary to our expectations, HTP users were most concerned about HTPs’ long-term safety.

Japanese physicians showed a low concern rate for HTP addiction. However, nicotine levels in HTP aerosols are approximately equivalent to those in conventional cigarettes.^11^ Previous studies showed that most HTP users concurrently smoked conventional cigarettes.^37^^,^^38^ The results suggested that Japanese physicians should pay more attention to HTP users and dual cigarette use.

Japanese physicians, particularly HTP users, were less concerned about HTP regulations than we expected. In Japan, tobacco regulation policies are far behind those in other countries, particularly in protecting individuals from passive smoking, including warnings on the tobacco package, sponsoring anti-tobacco mass media campaigns, and prohibiting tobacco advertising, promotion, and sponsorship.^39^ For example, the Health Promotion Law imposed more lenient regulations on HTP use in public places compared with conventional cigarettes.^40^ This could encourage smokers to use HTPs in certain spaces. Most HTP users in Japan smoked in indoor public spaces.^41^ Tobacco companies are attempting to rehabilitate their reputation so that they can more effectively influence governments or influencers to relieve existing tobacco regulation policies or create regulation exemptions.^42^ Rallying tobacco regulation policies will make it easier for companies to justify HTP use and increase social acceptability.^42^ Thus, Japanese physicians need to know the HTPs’ health effects, learn about tobacco companies’ marketing strategies, and understand current tobacco regulation policies. To acquire knowledge of HTPs, this study recommends the promotion of physician and public education campaigns through social network services or the mass media, with particular focus on the fact that HTPs are not less harmful than cigarettes, may cause passive smoking, and can serve as a gateway to conventional cigarettes.

Despite the important abovementioned findings, this study has several limitations. First, this survey was conducted with JMA members who have a higher average age compared to the overall Japanese physician population. Thus, it is possible that the prevalence of HTP smokers is underestimated, despite adjusting for age and gender. Second, as this study was cross-sectional, inferences on the causality of the relationships observed are limited and should be tentative. Third, as this was a self-administered survey, participants may have underestimated (or overestimated) smoking prevalence. In one study, it was found that women were more likely to underestimate the smoking prevalence compared to men.^43^ Fourth, our analysis excluded physicians who were not aware of HTPs, so the result may have overestimated the HTP concern rate among Japanese physicians. In addition, we did not investigate physicians’ knowledge of HTPs, which may affect their attitudes and concerns regarding HTPs. Therefore, future research should investigate the extent of this knowledge.

### Conclusion

Most participating physicians had awareness of HTPs. However, older physicians and certain specialty physicians, other than internal medicine specialists, lacked awareness. Even though a lack of evidence regarding HTPs’ long-term safety was perceived among Japanese physicians, they had fewer concerns about addiction and regulation. Physicians should receive training, so they are informed of new evidence of these products and tobacco regulation policies. Japan is a key battleground in the global fight against HTPs. If physicians’ attitudes toward HTPs are ambiguous, they cannot give adequate guidance to patients on how to stop smoking. If the number of HTPs users with or without cigarettes continues to increase, it would lead to rising trends of tobacco-related diseases. Physicians need to give guidance on tobacco cessation, including on HTPs, to ensure the well-being of patients. Japan’s experience suggests that policymakers should prohibit commercials and sales associated with HTPs and launch campaigns to inform individuals of the benefits and drawbacks of HTP use. Increasing evidence regarding HTPs’ health effects and educating physicians are critical first steps towards reducing tobacco-related deaths. Continued surveying is needed, including detailed knowledge of HTPs and how physicians should discuss HTPs with patients and their families.

## SUPPLEMENTARY MATERIAL

eTable 1. Rate and odds ratio of physicians’ awareness about HTP and e-cigarettes (N = 3,231)

eTable 2. Factors associated with physicians increasing awareness and discouraging the use of HTP among patients (N = 3,730)

eTable 3. Correlation of factors associated with HTP concerns among physicians with HTP awareness (N = 3,730)
