# Supplementary material for: Awareness, Attitudes, and Concerns Regarding Heated Tobacco Products Among Physicians in Japan
Source: J Epidemiol. 2023 Sep 5;33(9):441–9. doi: 10.2188/jea.JE20210470 (PMC10409525; doi:10.2188/jea.JE20210470)
Supplement: Supplementary file 1 [file je-33-441-s001.pdf]

eTable 1. Rate and odds ratio of physicians' awareness about HTP and e-cigarettes (N=3,231)

	Unweighted, Number	Weighted % (95% CI) [a]		P-value[b]	aOR	95% CI	P-value[c]
Total	3,231	67.2	[65.3–68.9]				
Sex							
Male	2,587	67.4	[65.4–69.4]	0.433	1.00		
Female	644	66.2	[62.1–70.1]		0.70	[0.54–0.89]	0.004
Age group, years							
24–39	192	78.8	[73.1–83.6]	<0.001	1.00		
40–49	614	72.5	[69.4–75.4]		0.68	[0.48–0.97]	0.036
50–59	975	66.4	[63.9–68.8]		0.49	[0.34–0.70]	<0.001
60–69	934	58.0	[55.5–60.4]		0.33	[0.23–0.48]	<0.001
≥70	516	40.4	[37.7–43.1]		0.16	[0.11–0.24]	<0.001
Smoking status							
Never smoker	2,166	67.6	[65.4–69.8]	<0.001	1.00		
Ever non-HTP smoker	803	62.6	[59.3–65.8]		0.99	[0.82–1.20]	0.948
Current non-HTP smoker	145	70.6	[60.7–78.8]		1.49	[0.93–2.39]	0.097
Ever HTP smoker	31	61.8	[34.8–83.1]		0.72	[0.21–2.48]	0.608
Current HTP smoker	86	86.9	[72.3–94.4]		2.67	[1.02–6.98]	0.045
Cessation discussion time							
None	666	55.0	[51.1–58.9]	<0.001	1.00		
<3 min	1,379	67.5	[64.6–70.3]		1.35	[1.08–1.69]	0.008
≥3min, <5min	752	75.5	[71.9–79.0]		2.13	[1.61–2.82]	<0.001
≤5min	395	74.1	[69.0–79.2]		2.15	[1.54–3.01]	<0.001
Medical department							
Internal medicine	1,689	70.3	[67.8–72.8]	<0.001	1.00		
Surgery & orthopedics	375	60.9	[55.4–66.4]		0.74	[0.55–0.99]	0.046
Other	1,159	65.0	[62.0–68.0]		0.89	[0.72–1.09]	0.259
Work form							
Owner	1,718	64.9	[62.6–67.2]	<0.001	1.00		
Employee	1,455	68.9	[66.2–71.7]		0.95	[0.74–1.21]	0.655
Facility							
Clinics	1,904	65.8	[63.6–68.0]	<0.001	1.00		
Hospitals	1,077	69.6	[66.4–72.9]		0.94	[0.72–1.24]	0.681
Other	133	57.0	[49.6–64.4]		1.10	[0.78–1.55]	0.255

aOR, adjusted odds ratio; CI, confidence interval; HTP, heated tobacco product.

[a] Weighted percentages were standardized according to the age group and sex composition of

physicians provided by Japan's Ministry of Health, Labour and Welfare.
[b] *P*-values were calculated by the analysis of variance.
[c] *P*-values were calculated by multivariate logistic regression.
[d] Odds ratios were adjusted for participants and statistics: population weight, sex, age group, smoking status, cessation discussion time, medical department, employment type, and facility.
[e] There were 48 respondents (1.3%) missing data on cessation discussion time, 10 respondents (0.3%) missing data on medical department, 80 respondents (2.1%) missing data on work form, and 143 respondents (3.8%) missing data on facility
[f] Missing data were imputed using multiple imputation by chained equations of 20 datsets.

eTable 2. Factors associated with physicians increasing awareness and discouraging the use of HTP among patients (N=3,730)

	Unweighted Number	Multivariate OR	95% CI	*P*-value
Health effects of HTPs				
Lack of evidence regarding the long-term safety of the product	2,480	1.15	[0.87–1.52]	0.332
Misguided assumption that HTPs are less harmful than cigarettes	1,940	1.73	[1.30–2.30]	<0.001
Addictive potential of HTPs				
Long-term health effects of nicotine addiction	1,493	1.55	[1.12–2.14]	0.009
HTP use may result in combined cigarette usage	321	0.75	[0.43–1.33]	0.331
Regulation of HTPs				
Lack of regulatory controls from the government	1,170	1.65	[1.17–2.33]	0.004
Function as attractive starter products and a gateway to smoking among young nonsmokers	1,504	1.27	[0.92–1.76]	0.145
Sex (compared to men)	2,964			
Women	766	0.84	[0.60–1.20]	0.342
Age group, years (compared to 24–39 years old)	221			
40–49	681	0.83	[0.55–1.25]	0.378
50–59	1,101	0.93	[0.62–1.40]	0.722
60–69	1,074	1.12	[0.73–1.71]	0.593
≥70	653	1.01	[0.64–1.60]	0.949
Smoking status (compared to never-use smoker)	2,484			
Ever-use non-HTP smoker	941	0.91	[0.68–1.23]	0.554
Current non-HTP smoker	165	0.22	[0.12–0.41]	<0.001
Ever-use HTP smoker	39	0.31	[0.11–0.89]	0.029
Current HTP smoker	101	0.39	[0.19–0.690	<0.001
Cessation discussion time (compared to none)	800			
<3 min	1,609	1.65	[1.19–2.30]	0.003
≥3min, <5min	841	1.74	[1.17–2.58]	0.007
≤5min	432	2.33	[1.51–3.60]	<0.001
Medical department (compared to internal medicine)	1,927			
Surgery and orthopedics	436	0.76	[0.49–1.17]	0.210
Others	1,357	0.70	[0.51–0.95]	0.022

CI, confidence interval; HTP, heated tobacco product.

[a] Multivariate odds ratios were adjusted for health effects of HTPs, the addictive potential of HTPs, regulation of HTPs, participants and statistics: population weight, gender, age group, smoking status, cessation discussion time, medical department, employment type, and facility.

[b] There were 48 respondents (1.3%) with missing data on cessation discussion time, and 10 respondents (0.3%) with missing data on the medical department.

[c] Missing data were imputed using multiple imputation by chained equations of 20 datsets.

eTable 3. Correlation of factors associated with HTP concerns among physicians with HTP awareness (N=3,730)

		1	2	3	4	5	6	7	8	9	10	11
Health effects of HTPs												
1	Lack of evidence regarding the long-term safety of the product	-										
2	Misguided assumptions that HTPs are less harmful than cigarettes	0.23	-									
3	Misguided assumptions that HTPs do not cause passive smoking	0.20	0.53	-								
Addictive potential of HTPs												
4	Long-term health effects of nicotine addiction	0.19	0.30	0.30	-							
5	HTP use may perpetuate smokers' addiction	0.20	0.43	0.41	0.44	-						
6	HTP use may result in combined cigarette usage	0.16	0.25	0.32	0.27	0.38	-					
Regulation of HTPs												
7	Lack of regulatory controls by the government	0.24	0.30	0.33	0.28	0.33	0.31	-				
8	Function as attractive starter products and a gateway to smoking for young nonsmokers	0.22	0.31	0.31	0.38	0.37	0.28	0.38	-			
9	Marketing and advertising of HTPs, especially to children and youth	0.18	0.34	0.35	0.37	0.44	0.38	0.37	0.48	-		
10	Become "bridge product" for use in places where smoking is prohibited	0.18	0.41	0.44	0.31	0.44	0.35	0.40	0.41	0.45	-	
11	Advertisement of HTPs by celebrities who vape will make cigarette smoking glamorous again and "renormalize" smoking	0.20	0.39	0.39	0.32	0.48	0.40	0.39	0.38	0.52	0.47	-

HTP, heated tobacco product.

[a] Data was measured using Spearman correlation coefficients.
